# Insights into PINK1/Parkin function and dysfunction from *Drosophila* models

**DOI:** 10.1042/BCJ20253459

**Published:** 2025-12-23

**Authors:** Seoyoung Park, Nikita Kozhushko, Thomas H. Wight, Alexander J. Whitworth

**Affiliations:** 1 MRC Mitochondrial Biology Unit, University of Cambridge, Cambridge Biomedical Campus, Cambridge, CB2 0XY, U.K.

**Keywords:** autophagy, calcium signalling, *Drosophila*, immune signalling, mitochondria, mitophagy, mtDNA, neurodegeneration, Parkin, Parkinson’s disease, PINK1

## Abstract

Loss-of-function mutations in *PINK1* and *PRKN* cause familial forms of Parkinson’s disease (PD). *In vitro* studies have revealed incredible insights into the molecular and cell-biological function of these genes, which have focused predominantly on mitophagy – the autophagic degradation of damaged mitochondria. The mechanisms of PINK1/Parkin function ultimately require investigation in an *in vivo* context using classic genetic approaches in animal models. In this context, *Drosophila* models have proven to be remarkably informative, in part due to robust phenotypes arising from null mutations. They have revealed important insights into the function of the Pink1 and parkin orthologues, much of which has proven to be conserved in humans. The simplicity, speed and genetic tractability make *Drosophila* an excellent *in vivo* model to interrogate the physiological functions of Pink1 and parkin and to rapidly test emerging hypotheses arising from *in vitro* work. They also represent a powerful model with which to explore the pathological consequences of *Pink1/parkin* loss in a whole-organism context. In this regard, several themes have emerged from recent studies that likely have significance for the neurodegenerative process in humans, including aberrant activation of immune signalling and consequent inflammation, disruptions to gut integrity and disturbed mitochondrial calcium handling. In this review, we evaluate the current evidence regarding the mechanism(s) of Pink1/parkin-mediated mitochondrial turnover in *Drosophila*, and discuss the potential implications of recent developments on the consequences of *Pink1/parkin* mutations and how these may inform the pathogenesis of PD.

## Introduction

Parkinson’s disease (PD) is characterised by progressive loss of dopaminergic (DA) neurons in the substantia nigra pars compacta (SNpc), leading to hallmark motor symptoms such as bradykinesia, resting tremor, muscle rigidity and postural instability. Although traditionally viewed as a motor disorder, it is now widely recognised that PD encompasses a broad spectrum of non-motor symptoms, including cognitive impairment, autonomic dysfunction and gastrointestinal (GI) abnormalities [1]. Currently, there is no cure due to an incomplete understanding of the pathogenic causes. Decades of research to understand the root cause of the disease have highlighted many pathogenic contributors including environmental and genetic factors. The success of genetic findings has yielded perhaps the most compelling insights into the pathogenic mechanisms. Mutations in a number of genes including *SNCA*, *LRRK2*, *VPS35*, *DJ-1*, *GBA*, *PRKN* and *PINK1* have been linked to inherited forms of PD, accounting for ~10% of cases, while genome-wide association studies (GWAS) have identified more than 90 risk loci for sporadic PD [2]. Experimental approaches to uncover both the normal function and pathogenic dysfunction of these genetic factors are crucial to understanding the molecular causes of PD. Such studies have consistently highlighted several mechanisms, including proteostasis and autophagy-lysosomal degradation, mitochondrial dysfunction, immune signalling and inflammation [3,4].

Despite these advances, major questions remain around the selective vulnerability of SNpc DA neurons. Leading hypotheses point towards several physiological features, such as their extensive axonal arborisation and autonomous pacemaking activity, which confer a substantial bioenergetic and metabolic burden [5–7]. Consistent with this, multiple lines of evidence implicate mitochondrial dysfunction as a key driver of neurodegeneration, from both environmental and genetic causes [8,9]. Indeed, the identification of loss-of-function mutations in *PINK1* and *PRKN* – which encode PINK1 (a mitochondrially targeted serine/threonine kinase) and Parkin (a cytosolic E3 ubiquitin ligase), respectively – as a cause of autosomal recessive, early-onset PD has cemented mitochondrial disruptions as a major contribution to PD [10,11].

Since their linkage to PD more than 20 years ago, significant progress has been made in elucidating the molecular and cellular functions of PINK1 and Parkin. Today, they are best known for their role in mitophagy – a key mitochondrial quality control (MQC) process, important for the homeostatic regulation of the mitochondrial network, whereby PINK1 acts in conjunction with Parkin to degrade damaged mitochondria via the autophagy–lysosome pathway. While macroautophagy is an essential cellular process that delivers cytoplasmic components in bulk to lysosomes for degradation (mediated by the large family of ATG proteins), mitophagy specifically targets the engulfment and degradation of mitochondria, usually in the context of removing damaged organelles. The current state of knowledge of PINK1/Parkin-mediated mitophagy has recently been comprehensively reviewed [12,13], so the key aspects of this mechanism are briefly summarised as follows.

In healthy mitochondria, PINK1 is partially imported, proteolytically processed by PARL and other proteases, and returned to the cytosol for proteasomal degradation. Upon mitochondrial damage, PINK1 becomes stabilised on the outer mitochondrial membrane (OMM), where it phosphorylates Parkin and ubiquitin bound to OMM proteins, on Serine-65 of both proteins. Phosphorylation of Parkin stimulates its ligase activity, allowing it to ubiquitinate numerous OMM proteins. This, in turn, provides additional substrate for PINK1-mediated phosphorylation driving a feedforward mechanism, ultimately resulting in dysfunctional mitochondria being decorated with phospho-Serine 65 ubiquitin (pS65-Ub) chains on their outer surface. These chains serve as a signal for the recruitment of autophagy machinery and trigger subsequent engulfment of the damaged mitochondria by autophagosomes. The exact mechanisms by which this recruitment takes place are still debated, however [14]. Some have proposed phospho-ubiquitin to act as a receptor for the adaptor proteins, including OPTN and NDP52 [15], while others have suggested that it is the unphosphorylated form that is preferentially responsible for this initiation [16,17]. While these molecular details remain to be resolved, ultimately, autophagosome-sequestered mitochondria are subsequently trafficked to lysosomes for degradation. Thus, mitophagy is considered to be critical for the clearance of damaged mitochondria, thereby preventing disruptions in cellular homeostasis, especially in energy-demanding tissues such as the brain and muscle.

**Figure 1:**
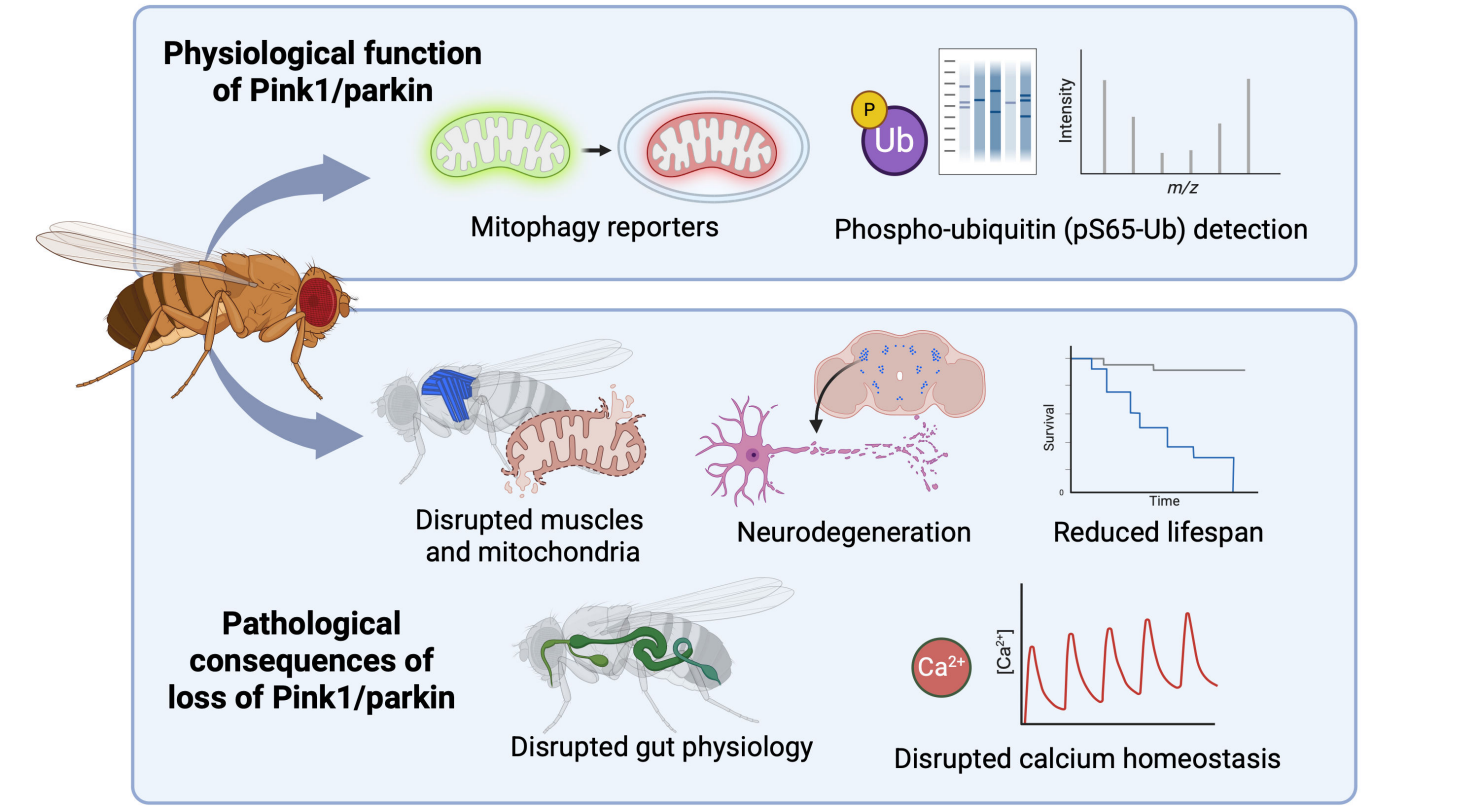
*Drosophila* as a useful model system to study Pink1/parkin-mediated pathways. **Top**: The *in vivo* role of Pink1/parkin in mitophagy has been characterised in *Drosophila* using fluorescence-based mitophagy reporters, together with phospho-ubiquitin detection via immunoblotting and mass spectrometry. **Bottom:** The pathological consequences of Pink1/parkin loss are also readily interrogated in *Drosophila* owing to the presence of several robust and quantifiable phenotypes, typically in high energy-demanding tissues.

For many years, *Drosophila melanogaster* models have been instrumental in illuminating our understanding of the basic biology of PINK1 and Parkin as well as the downstream consequences of their dysfunction (Figure 1). *Drosophila* has clear orthologues of the *PINK1* and *PRKN* genes (here referred to as *Pink1* and *parkin*, following FlyBase nomenclature, with the corresponding protein names, Pink1 and parkin, to differentiate them from their mammalian counterparts). Early studies of *Pink1/parkin* mutants revealed an array of striking phenotypes including locomotor defects (commonly assessed via negative geotaxis or ‘climbing’ assays), reduced lifespan, profound mitochondrial disruptions, particularly in flight muscles, male sterility, and (modest) DA neurodegeneration [18–21]. It was these studies that provided the foundational observations that Pink1 and parkin play a critical role in maintaining mitochondrial integrity *in vivo*, particularly in high energy-demanding tissues. Notably, this was in stark contrast to the *Pink1/Prkn* knockout (KO) mice that showed little neuropathology, motor phenotypes or mitochondria dysfunction [22–26], limiting their utility to study PINK1/Parkin-associated pathways.

**Figure 2:**
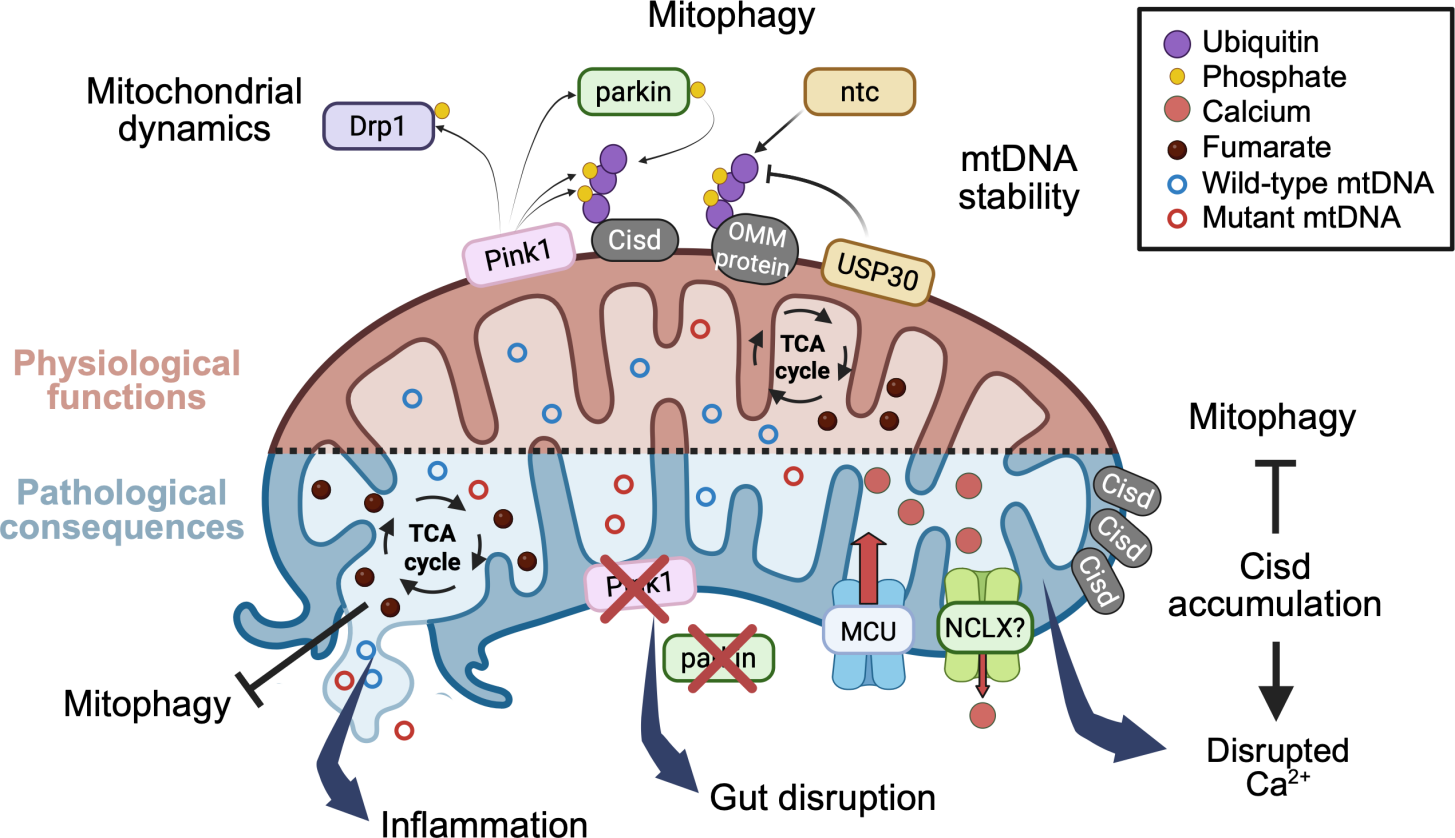
Physiological functions and pathological consequences of the loss of Pink1/parkin. Overview of recent advances in *Drosophila* research on Pink1/parkin function, as discussed in this review. **Top**: Pink1/parkin play a crucial role in multiple mitochondrial quality control (MQC) processes. In *Drosophila*, as in humans, Pink1 phosphorylates both ubiquitin and parkin, promoting parkin-dependent ubiquitination of several outer mitochondrial membrane (OMM) proteins, including Cisd, leading to subsequent mitophagy. Recent work in *Drosophila* and other models has highlighted key regulatory factors influencing this pathway: USP30, which counteracts parkin activity through deubiquitination; ntc, which enhances basal mitochondrial ubiquitination; and the TCA cycle metabolite, fumarate has been implicated in mitophagy inhibition, although its precise mechanism in *Drosophila* remains unclear. Beyond mitophagy, Pink1/parkin regulate mitochondrial dynamics through Pink1-mediated phosphorylation of the mitochondrial fission factor Drp1 and contribute to the maintenance of mtDNA integrity by limiting mutation load via an autophagy-dependent pathway. **Bottom**: Loss of Pink1/parkin disrupts MQC, leading to the activation of multiple downstream pathways linked in PD pathogenesis. Defective MQC promotes the release of mitochondria damaged-associated molecular patterns (mtDAMPs) that trigger inflammatory responses, although the specific mtDAMP species and innate immune signaling pathway(s) involved in *Drosophila* remain to be defined. In parallel, Pink1/parkin deficiency perturbs mitochondrial Ca^2+^ handling via the MCU and NCLX, a defect thought to be further exacerbated by age-dependent accumulation of the parkin substrate, Cisd, by impacting ER Ca^2+^ flux and inhibiting mitophagy. Additional consequences of MQC disruption may include altered fumarate levels and increased mtDNA mutation load, although these potential effects require further investigation.

Subsequently, many studies of the *Drosophila* models have provided important insights into the conserved mechanisms of PINK1/Parkin function and, indeed, laid the foundation for the *in vitro* studies that have described the molecular details of the PINK1/Parkin mitophagy pathway. For instance, genetic interaction studies first established that *Pink1* and *parkin* act in a common pathway with Pink1 acting upstream of parkin [20,21]. Subsequent hypothesis-driven experiments identified the fly orthologue of PARL, rho-7, as the intramembrane protease that cleaves Pink1 [27], while a combination of genetic screens and hypothesis testing revealed a striking interaction of Pink1/parkin with the mitochondrial dynamics machinery [28,29], and molecular studies identified the Mitofusin orthologue, Marf, as a Parkin substrate [30]. *Drosophila* models also provided the first compelling *in vivo* evidence that Pink1 and parkin regulate mitochondrial turnover under physiological conditions [31]. These and other early studies of the *Drosophila* Pink1/parkin models have been thoroughly reviewed before [32–34]; therefore, this review will focus on highlighting recent advances in understanding Pink1/parkin function and dysfunction in *Drosophila*. In particular, we focus on three main aspects: (i) the manifestation and regulation of mitophagy under physiological conditions; (ii) the involvement of aberrant immune signalling in Pink1/parkin pathology and the potential involvement of a gut-brain axis; (iii) the intersection of Pink1/parkin with intracellular calcium (Ca^2+^) flux (Figure 2 *).*

### Molecular mechanisms of Pink1/parkin mitophagy in *Drosophila*

In recent years, remarkable progress has been made in elucidating the molecular and cell-biological mechanisms of PINK1/Parkin-mediated mitophagy – in part due to the relative ease of studying this in classic *in vitro* cell models, such as HeLa, HEK293 or similar cell lines, where chemical agents that disrupt the mitochondrial membrane potential robustly activate PINK1 and initiate the cascade of downstream events. While this has been a useful experimental approach to provoke severe mitochondrial dysfunction and acute stimulation of PINK1, this, along with the use of Parkin overexpression in cells that lack it (e.g. HeLa cells), likely represents an exaggerated or accelerated response compared with more subtle or transient mitochondrial perturbations that typically occur *in vivo*. Moreover, immortalised cell lines have quite different metabolic profiles than *in vivo* tissues, often growing in glycolytic conditions where mitochondria are minimally required and experiencing quite different oxygen tension [35]. Thus, it is inevitable that these *in vitro* findings require evaluation *in vivo*, partly to confirm that these mechanisms operate similarly in an *in vivo* context and to investigate nuances such as tissue-specific differences, but also to determine how they relate to more physiologically relevant stimuli. Encouragingly, several key features of the mammalian PINK1/Parkin signalling pathway are clearly conserved and detectable in *Drosophila*: the accumulation of Pink1 on the OMM of dysfunctional mitochondria and the consequent recruitment of parkin [30,36]; the Pink1-dependent phosphorylation of both parkin and ubiquitin [37–40]; and the recruitment of Ref2)p (the fly orthologue of p62/SQSTM1) and Atg8a (the fly orthologue of LC3) to dysfunctional mitochondria [41,42].

It is important to note, however, that several key differences exist between *Drosophila* and mammalian systems. For instance, while mammalian PINK1 is very rapidly cleaved and degraded under basal conditions, typically requiring strong inhibitors of mitochondrial function and/or degradation pathways to detect it [43], both cleaved and full-length *Drosophila* Pink1 is detectable under steady-state conditions [27,44,45]. Turnover of *Drosophila* parkin substrates also appears to follow different kinetics as they are often seen to accumulate under steady-state conditions in *Drosophila Pink1/parkin* mutants, whereas they do not in mammalian models [46–48]. In fact, given what is now known about the molecular signalling mechanisms of mammalian PINK1 and Parkin, it remains unclear why *parkin* overexpression is sufficient to suppress *Pink1* phenotypes in *Drosophila* (as seen in the original papers genetically linking *Pink1* and *parkin* [20,21]), while Parkin overexpression in cultured cells is not sufficient to induce mitophagy [43,49]. The reasons for these differences are not currently known but likely reflect a combination of different relative requirements for PINK1/Parkin-mediated MQC, with possibly greater activity in *Drosophila* consistent with stronger loss-of-function phenotypes, and/or more complex mechanisms in mammals.

Since the phosphorylation of ubiquitin is a key conserved (and, as far as we know, unique) function of PINK1, antibodies that robustly detect pS65-Ub have opened up the possibility to follow the spatiotemporal activity of PINK1 under different experimental conditions. Due to the deep conservation of these molecules, antibodies raised against mammalian pS65-Ub also work well in *Drosophila* [39,40]*.* Under steady-state conditions, pS65-Ub is extremely lowly abundant in young, wild-type *Drosophila* tissues such that it is essentially undetectable by immunoblotting [39,40]*.* However, pS65-Ub is detectable in young flies by sensitive mass spectrometry combined with ubiquitin enrichment methods [40]. The very low level of pS65-Ub is consistent with PINK1 activation being triggered by mitochondrial damage or stress – presumably a rare event in young, healthy animals. Also consistent with this notion, pS65-Ub levels increase with age, becoming robustly detectable by mass spectrometry after substantial ageing, presumably triggered by accumulated mitochondrial damage or dysfunction [40]. It will be interesting to investigate in more detail the dynamics of pS65-Ub production with age and other physiologically relevant stressors such as dietary challenges, exercise, infection or sleep deprivation.

Strikingly, pS65-Ub was also found to accumulate to high levels in *parkin* mutant flies, detectable by immunoblotting, immunostaining and mass spectrometry [39,40]. Moreover, the profound disruption to muscle tissue in *parkin* mutants allowed researchers to show for the first time *in vivo*, by immunofluorescence and immuno-electron microscopy (EM) analysis, that pS65-Ub specifically accumulated on disrupted mitochondria [40]. It is interesting to note that while pS65-Ub also modestly accumulates in mouse tissue lacking Parkin [50], this occurs predominantly in heart, which is structurally very similar to *Drosophila* flight muscle due to the high mitochondrial content. The accumulation of pS65-Ub in *parkin* mutants is consistent with parkin being required to mediate mitophagy downstream of ubiquitin phosphorylation: Pink1 can be activated but pS65-Ub and, hence, pUb-labelled mitochondria are not subsequently degraded in the absence of parkin. On the other hand, it also demonstrates that pS65-Ub on mitochondria is not sufficient by itself to mediate mitophagic degradation. Nevertheless, detection of pS65-Ub provides a molecular readout with which to investigate the downstream mechanism of degradation *in vivo*. This was greatly facilitated by the finding that pS65-Ub rapidly accumulates upon systemic exposure (i.e. feeding) of the oxidative stressor, paraquat, which provided an opportunity for pulse-chase analysis to follow the degradation mechanisms [40]. Surprisingly, in contrast with the canonical view of mammalian PINK1-Parkin pathway, removal of the key autophagy gene *Atg5* only partially blocked pS65-Ub turnover in *Drosophila* [40], which was mirrored by inhibition of the proteasome, indicating both degradation pathways are required. These findings align with some results from mammalian cell culture systems which indicate that proteasome activity is required for Parkin-mediated mitophagy [51], and other studies propose degradation occurs in a piecemeal fashion via mitochondria-derived vesicles (MDV) trafficking directly to lysosomes [52–55]. The small (sub-organellar) scale and transient nature of MDVs render them hard to study, especially *in vivo*. Consequently, little is currently known about the complexities of their formation, dynamics and cargo selectivity. But, as with many areas of molecular cell biology, the tractability of *Drosophila* may prove informative here.

Consistent with a more piecemeal, selective degradation process, an early study analysing the turnover rate of mitochondrial proteins in *Drosophila* using stable isotope-labelling and mass spectrometry provided the first direct evidence that Pink1/parkin promote mitochondrial turnover *in vivo* and revealed a surprising selectivity to this [31]. Mitochondrial protein turnover was reduced in *parkin* mutants to a similar extent as *Atg7* mutants, which is consistent with autophagic degradation but does not directly prove it as this could also occur via a different mechanism. Interestingly, differentially degraded proteins were enriched for membrane-bound components of the respiratory chain. Curiously, *Pink1* mutants showed a more modest overall impact on mitochondrial protein turnover, although the most affected proteins were still membrane components of the respiratory chain. Furthermore, a subsequent study by Pallanck’s group found that around one-third of mitochondrial protein turnover was via autophagy under steady-state conditions, indicating that most mitochondrial turnover occurs via other mechanisms [56]. This unexpected degradation pattern revealed by the *parkin* mutants differs from the *in vitro* model of wholesale engulfment and bulk degradation and indicates that a more selective process takes place *in vivo* favouring membrane respiratory chain proteins. While this analysis specifically described basal turnover in relatively young flies, without further stimulus or stress induction, the stimulus for Pink1/parkin activity in young flies is unclear, and it remains to be determined whether this occurs similarly upon ageing or other pathogenic conditions. Nevertheless, these findings are complemented by evidence from human induced pluripotent stem cell (iPSC)-derived neurons which also supports selective Complex I protein engulfment by PINK1/Parkin-positive autophagosomes in a model of MELAS syndrome [57]. Clearly, further work is needed to better understand the mechanistic details of Pink1/parkin-mediated mitochondrial degradation *in vivo* and the *Drosophila* models offer a tractable system to untangle this.

Together, these studies reveal that while *in vitro* approaches using strong depolarising agents have illuminated the pathway’s capacity for bulk mitophagy, the physiological mechanisms in *Drosophila* likely involve more selective, piecemeal turnover, possibly via alternative routes such as vesicular trafficking pathways. This highlights the need for a thorough investigation of these mechanisms across the complexity of *in vivo* contexts.

### Analysing and visualising mitophagy *in vivo*

A highly informative advance to the field came from the development of a number of fluorescence-based mitophagy reporters, designed to reveal the presence of mitochondrial content in the lysosome. To date, the most commonly used are mt-Keima and mito-QC/mtx-QC, but others have been reported including mito-SRAI and mt-Rosella [58–62]. While these reporters differ in their molecular details, they all exploit the relative acidity of lysosomes to elicit a fluorescence spectral shift when the reporter enters the acidic environment. It is also important to note that they can be targeted to different mitochondrial sub-compartments (OMM for mito-QC, matrix for the others), which has implications for the inference of which mitochondrial components are being reported.

Lee et al. [63] first reported the mitophagy sensors, mito-QC and mt-Keima, in *Drosophila* and observed that basal mitophagy (i.e. under steady-state conditions) is widespread across many tissues and developmental stages, indicating a homeostatic rate of mitochondrial turnover. This initial study reported the surprising observation that loss of *Pink1* or *parkin* had almost no effect on basal mitophagy across a range of tissues, developmental stages and adult ages, even in tissues with clear *Pink1/parkin* phenotypes. These results were in broad agreement with a contemporaneous study analysing mito-QC in *Pink1* KO mice [64]. In contrast, a subsequent study by Cornelissen et al. [65], using mt-Keima, reported that mitophagy in flight muscles increased markedly with age but was abrogated upon *Pink1* or *parkin* loss, and basal mitophagy in DA neurons was partially blocked by *parkin* knockdown. While the main conclusions of these studies appear discordant, it is worth considering the experimental context in greater detail. For instance, in *Pink1/parkin* mutants, the flight muscle is well documented to have undergone apoptosis early in adult life [18,21], so while remnants of the tissue may remain, interpretations of subtle cell biology in the remaining tissue should be made with care. Similarly, for DA neurons, some of which degenerate in *Pink1/parkin* mutants, caution should be exercised when analysing aged and potentially degenerating neurons, especially when live-cell imaging as required with mt-Keima.

It is worth mentioning that the relative sensitivity of the different mitophagy reporters has been debated in the literature [66,67] and used to question the results from flies and mice using mito-QC, but this does not satisfactorily account for the contrasting results. For instance, although Lee et al. performed the majority of experiments with mito-QC, both mito-QC and mt-Keima reporters were initially analysed in parallel with similar results. While Lee et al. did not detect Pink1/parkin-mediated mitophagy in adult tissues, a recent study successfully detected mitophagy in the *Drosophila* germline using mito-QC but also found this to be independent of Pink1 or parkin [68]. Equally, both the mito-QC and mt-Keima reporters have been used to reveal Pink1/parkin-dependent mitophagy, in fly intestines during metamorphosis [69] or upon rotenone or hypoxia treatment [70]. While the latter study aligns well with the paraquat-induced pS65-Ub accumulation discussed earlier [40], the former study by Shen and colleagues provides an important new paradigm for analysing mitophagy. First, this study complemented the mitophagy flux analysis using mito-QC with transmission EM as an orthogonal approach to demonstrate mitophagy, which emphasises the importance of using multiple approaches. Second, the cellular context (tissue remodelling during metamorphosis) represents a physiologically relevant mitophagy, akin to organelle clearance from erythrocytes [71], which highlights that mitophagy quite likely occurs differently in different cellular contexts.

Considered together, analysis of the mitophagy reporters, pS65-Ub dynamics and mitochondrial turnover rates presents a complex picture. The weight of evidence from mitophagy reporters (in flies, mice and cultured cells) indicates that basal mitophagy is not predominantly via a Pink1/parkin-mediated mechanism, although this can clearly be provoked by toxic stimuli and possibly by age-related stresses. Nevertheless, proteomic analyses indicate that Pink1/parkin do mediate a selective form of protein turnover under basal conditions, although the mechanism of this turnover is currently unclear. On the other hand, the selectivity in Pink1/parkin-mediated mitochondrial protein turnover coupled with the pattern of pS65-Ub degradation suggests that the Pink1/parkin pathway supports mitochondrial quality control via mechanisms extending beyond classic bulk mitophagy, which may also be highly context specific.

Overall, these studies have delivered fundamental new insights into how PINK1/Parkin may work *in vivo* and begun to challenge previous assumptions. Ultimately, the current lack of consensus underscores our current poor understanding about where, when and under what stimuli PINK1/Parkin mitochondrial degradation is activated and/or necessary. Determining the physiological context and mechanism of PINK1/Parkin-mediated MQC remains critical for understanding how mitochondrial dysfunction is managed in ageing and how it contributes to neurodegenerative disease. The methods available in *Drosophila* position these models well for addressing these questions.

### Uncovering regulators of mitophagy

Beyond the core PINK1/Parkin machinery, a growing body of work has uncovered additional regulators that fine-tune MQC processes and influence the recruitment or efficiency of this pathway. Understandably, there is intense interest from across academia and industry to uncover the mechanisms behind various molecular effectors that regulate mitophagy to identify actionable targets as a therapeutic strategy against mitochondrial dysfunction. One common class of proteins that has garnered attention is the deubiquitinases (DUBs). The rationale to inhibit DUBs is based on targeting mechanisms that could negatively impact Parkin-mediated ubiquitination. Consequently, USP30 has emerged as a prominent antagonist of Parkin substrate ubiquitination and inhibitor of mitophagy [72]. Early studies showed that depletion of USP30 in *Pink1* or *parkin* mutant flies suppressed many of the phenotypes including mitochondrial and flight muscle integrity, DA neurodegeneration and climbing ability [73]. Subsequent studies firmly established that USP30 is capable of counteracting Parkin-mediated ubiquitination *in vitro* [74,75]*,* yet until recently, it was unclear which Ub-ligase USP30 was counteracting in flies, since *USP30* loss still suppressed the mutant phenotypes in the absence of *parkin* [73]. An investigation into the function of *Drosophila ntc* (the fly orthologue of another PD-linked E3 Ub-ligase *FBXO7*) showed that ntc/FBXO7 mediates the basal mitochondrial ubiquitination required for PINK1-mediated phosphorylation and subsequent mitophagy [76]. Consequently, genetic analyses revealed that the loss of *USP30* derepresses ntc/FBXO7-mediated ubiquitination to promote mitophagy in *Pink1/parkin* mutants. While the homeostatic functions of USP30 are still emerging, including as a QC regulator of mitochondrial protein import [77], the potential of USP30 inhibition as a therapeutic target is also supported in additional models of PD [78]. Potent small molecule inhibitors targeting USP30 have been identified [79–81] and are now progressing in clinical trials*.*

The potential impact of other DUBs has inevitably garnered attention in the context of PINK1/Parkin-mediated mitophagy. Studies using *Drosophila* have shown that genetic reduction of USP8, USP14 and CG8334 (which has homology to both USP15 and USP32) all confer variable suppression of *Drosophila Pink1/parkin* phenotypes [82–84]. While the genetic and pharmacological inhibition of USP8 was shown to act by restoring the steady-state levels of the parkin substrate Marf and promote parkin-independent mitophagy *in vivo* [82], the mechanisms underlying the phenotypic suppression by the other DUBs *in vivo* remain unclear*.*

Mitophagy as a QC process is generally considered in the context of a homeostatic regulation of mitochondrial function and, hence, overall cellular metabolic state. Thus, it is rational that mitophagy may be activated by changes in metabolic conditions and even specific metabolites. In this regard, the *Drosophila* models have also been informative in identifying metabolic regulators of mitophagy *in vivo*. Early transcriptomic and metabolomic studies of *Pink1* mutants indicated that one-carbon, nucleotide and folate metabolism were disrupted and that genetic or dietary increase in these molecules could suppress many *Pink1* and *parkin* neurodegenerative phenotypes [85,86]; however, it remains unknown whether this occurs via modulating mitophagy. In contrast, a recent study has implicated the TCA cycle metabolite fumarate in directly inhibiting Parkin-mediated mitophagy [87]. In a mini-screen of metabolic genes, reduction of fumarate hydratase (FH), which leads to elevated levels of fumarate, was identified as a strong inhibitor of mitophagy. Fumarate was found to covalently modify (i.e., succinate) two cysteine residues on human Parkin (C323 and C451), preventing its activation and mitochondrial recruitment during stimulated mitophagy. Interestingly, C323 and C451 are not conserved in *Drosophila* and accordingly *Drosophila* parkin is insensitive to succination. Nevertheless, fumarate levels increase with age in flies and mammals, supporting the notion that age-related dysregulation of fumarate metabolism may contribute to impaired mitophagy. It is also interesting to reflect that loss of FH can induce the release of mitochondrial DNA (mtDNA) into the cytosol, activating innate immune responses [88], which may be a key driver of pathology upon loss of mitophagy (see below).

In addition to direct metabolite effects, metabolic pathways and organelles that interface with mitochondrial homeostasis have been implicated. For example, lipid droplets (LDs) have recently been shown to promote efficient mitophagy. In mammalian systems, DGAT1-dependent LD biogenesis stimulated by iron depletion was found to support lysosomal activity and mitochondrial turnover, while in *Drosophila*, loss of the *DGAT1* orthologue, *midway*, impaired neuronal mitophagy and locomotor performance. This suggests that metabolic rewiring toward lipid storage can directly impact MQC [89].

In a similar vein, *Drosophila fumble* (*fbl*), the orthologue of human *PANK2*, which catalyses the initial, rate-limiting step of *de novo* Coenzyme A (CoA) synthesis, was shown to genetically interact with *Pink1* [42]*.* Noting that CoA and acetyl-CoA were reduced in *Pink1* mutants [85], and that loss of *fbl* caused similar mitochondrial and neurodegenerative phenotypes, *fbl* overexpression restored CoA/acetyl-CoA levels and suppressed *Pink1* phenotypes. Furthermore, dietary supplementation with vitamin B5 derivatives (a substrate for CoA synthesis) also restored CoA/acetyl-CoA levels and suppressed phenotypes in *Pink1* mutants. Mechanistically, *fbl* overexpression was proposed to promote mitophagy by enhancing the acetylation of Ref(2)p. Given this, it is curious that *fbl* did not genetically interact with *parkin* or suppress any *parkin* phenotypes, although whether *fbl* overexpression could still induce mitophagy in the absence of *parkin* was not investigated. This study points to another potential therapeutic intervention strategy in PD treatment, although the impact of Fbl/PANK2 on mitophagy regulation requires more complete understanding.

The studies discussed here, and many others, highlight the utility of *Drosophila* for investigating the complexities of PINK1-Parkin mitophagy. Nevertheless, it is important to acknowledge some known limitations. While the core components of the PINK1/Parkin pathway are conserved, flies lack clear homologues of several mammalian mitophagy adaptors, such as NDP52 and TAX1BP1, with an unconfirmed orthologue of OPTN, all of which link ubiquitinated mitochondria to the autophagy machinery via LC3 binding [90]. In HeLa cells, PINK1-Parkin mitophagy occurs primarily through NDP52 and OPTN [15], whereas this is mediated by Ref(2)p/p62 in *Drosophila* [91]*.* Thus, mitophagy proceeds through partially divergent mechanisms downstream of PINK1 activation. Whether additional adaptors exist in flies or whether autophagy machinery is directly recruited in a Parkin-independent manner remains to be established. Approaches such as live-imaging of mitophagosome formation, genetic interaction screens and EM-based mapping of autophagic structures in *Drosophila* would be valuable to fill these gaps. It is worth noting that in recent years, advanced cellular models such as iPSC-derived neurons have added considerably to the field by illuminating cell type specific differences in temporal dynamics of the PINK1/Parkin machinery, such as Parkin-specific ubiquitylation patterns [75], in a physiologically relevant cellular context. These models can provide an important complement to *in vivo* studies.

## PINK1/Parkin in other mitochondrial quality control processes

Early studies in *Drosophila* established a genetic link between *Pink1/parkin* and regulators of mitochondrial dynamics which, alongside contemporaneous studies [49,92], cemented this cell-biological mechanism as an integral part of the mitophagy process. Recent investigations have continued to uncover the varied interactions of PINK1/Parkin with mitochondrial dynamics factors, with genetic analyses in the fly models providing important *in vivo* validation. Several studies initially linked fly Pink1/parkin to regulating the ubiquitination and steady-state levels of the Mitofusin orthologue, Marf, inhibiting re-fusion of dysfunctional mitochondria [93]. Now, recent studies have revealed multiple links to Pink1/parkin regulating mitochondrial fission via Drp1.

Human PINK1 was found to phosphorylate Drp1 at S616 promoting its pro-fission activity [94], and genetic approaches in *Drosophila* demonstrated that expression of phosphomimetic Drp1^S616D^ suppressed *Pink1* phenotypes. Surprisingly, the suppression occurred even in ‘autophagy-deficient’ (*Atg7* null) flies, suggesting that this was not mediated via a canonical mitophagy mechanism. However, it is important to note that authoritative guidance on autophagy recommends that conclusions should not be based on analysis of a single *Atg* gene [95]. A relevant example in this context is the finding that autophagy is necessary for mitochondrial clearance and cellular remodelling in the *Drosophila* intestine (midgut) during metamorphosis. However, neither Atg7 nor Atg3 is required for this process [96].

Curiously, the cyclin-dependent kinase CDK8 was also found to phosphorylate Drp1^S616^, and overexpression of fly Cdk8 also suppressed *Pink1* mutant phenotypes, suggesting a common mechanism [97]. Interestingly, using the powerful ‘GeneSwitch’ technique that allows temporal transgene induction, overexpression of Drp1 from midlife onwards (or even for a short period in midlife) is sufficient to significantly improve mitochondrial function and extend *Drosophila* lifespan [98]. Although studied for many years, new regulators of mitochondrial dynamics are still being identified [99], such as phosphatidylinositol-4 kinase IIIβ promoting fission downstream of Drp1 [100], originally identified in a screen for *Pink1/parkin* mitochondrial morphology modulators in *Drosophila* cells [101]. Consistent with this, the *Drosophila* gene encoding phosphatidylinositol-4 kinase, *four wheel drive* (*fwd*), similarly affects mitochondrial dynamics and overexpression was sufficient to suppress *Pink1/parkin* phenotypes. Moreover, loss of *fwd* prevented the suppression of *Pink1/parkin* mutants by *Drp1* overexpression, confirming that Fwd promotes mitochondrial fission downstream of Drp1 *in vivo* [102] as it does *in vitro* [100]*.*

It is now well-recognised that multiple pathways promote mitophagy besides PINK1/Parkin, including BNIP3/NIX and FUNDC1 (reviewed in [90]). Thus, it is important to understand the extent to which these alternative mitophagy pathways are conserved in *Drosophila*, to evaluate the extent to which they are actually independent pathways, and to investigate whether they can be co-opted to substitute for loss of *Pink1* or *parkin in vivo*. To date, no studies have systematically analysed the fly orthologues of *BNIP3* or *FUNDC1* in depth; however, studies have started to evaluate their role in contextual mitophagy. One study combining fly and cell models found that loss of the DUB, UCHL1, induced mitophagy in cells and was able to suppress *Pink1/parkin* fly phenotypes in a FUNDC1-dependent manner [103]. Although compelling evidence correlated mitophagy induction in cells with suppression of *Pink1/parkin* phenotypes, it was not formally proven that the suppression in flies occurred via mitophagy. Indeed, a separate study showed that overexpression of human FUNDC1 suppressed *Pink1* mutant locomotor, muscle and mitochondrial phenotypes, but this likely did not occur via mitophagy as it did not require the canonical LC3-binding domain or the autophagy machinery [104]. Instead, this occurs via a Drp1-dependent mechanism, suggesting that FUNDC1 acts to promote mitochondrial fission. This study adds further support to the long-standing view that a key driver of the *Pink1/parkin* phenotypes in *Drosophila* is due to disrupted mitochondrial dynamics which itself influences the efficacy of mitochondrial turnover.

On the other hand, emerging evidence supports that BNIP3 does mediate some mitophagy in *Drosophila* since loss of BNIP3 prevents mitochondrial clearance during the remodelling of fly intestines [44]. Moreover, additional pathway components are likely to be conserved since the overexpression of human BNIP3 also induces neuronal mitophagy in flies, increasing lifespan and improving old-age vitality [105], and suppressed neuromuscular phenotypes in a fly model of *Gdap1* loss [106]. It is currently unknown whether BNIP3 expression is sufficient to compensate for *Pink1/parkin* loss. Indeed, the functional relationship between PINK1/Parkin-mediated versus BNIP3-mediated mitophagy is poorly characterised *in vivo*, and the relative contributions of these pathways may be quite different in different tissues. Notably, Pink1, parkin and BNIP3 all promote mitophagy in the fly intestine [44,69], which may provide a good model system to thoroughly evaluate their interplay.

### PINK1/Parkin mitophagy and the regulation of mtDNA mutations

A unique feature of mitochondria as a metazoan organelle is that they have their own small genome (mtDNA), encoding several components of the OXPHOS machinery [107]. MtDNA is vulnerable to mutation from mitochondrial reactive oxygen species (ROS), and with limited repair mechanisms exemplifies a ‘Müller’s ratchet’ vicious cycle leading to potentially catastrophic damage if unmanaged [108,109]. Notably, mtDNA mutations accumulate in SNpc neurons with normal ageing and to remarkably high levels in individuals with PD [110,111]. Thus, PINK1/Parkin mitophagy as a MQC process has obvious implications for managing mtDNA mutations.

Initial investigations in mammalian cultured cells found that Parkin overexpression selectively decreased levels of pathogenic mtDNA variants, with long-term expression capable of nearly eliminating deleterious *MT-COI* mutations [112]. While this effect could be partly mediated by elimination of less healthy cells during replication, it was supported by subsequent observations in mice that showed locomotor deficits and DA neurodegeneration arise in *Prkn* KO mutants when combined with a strain that introduces stochastic mtDNA mutations (*PolgA* ‘mutator’) [113]. However, two subsequent studies failed to replicate these results [114,115]. Nevertheless, a similar approach using *Drosophila* bearing a mtDNA deletion did show evidence that *Pink1* and *parkin* overexpression reduced the mutation load in indirect flight muscles [116]. This selection appeared to be autophagy-dependent, as the beneficial effect was lost in flies lacking Atg8a, and importantly, the selectivity was specific to the deletion-bearing genomes rather than affecting total mitochondrial mass non-specifically. Complementary results were also seen in an analogous *C. elegans* model [117,118], supporting a purifying role of Pink1 and parkin against mtDNA mutations. Interestingly, in the context of purifying selection during programmed germline mitophagy, neither Pink1 nor parkin appear to play a role [119,120]; instead, this process is driven by BNIP3-mediated mitophagy [68,120]. Together, these findings show that mtDNA mutation recognition and clearance is a complex process that can occur through a number of different mechanisms which depend on the temporal and tissue-specific context. It will be of significant translational interest to determine whether mechanisms operating in one context (e.g. somatic versus germline selection) can be co-opted to perform in another as a potential therapeutic approach.

Interestingly, a recent targeted screening approach using a *Drosophila* model with proofreading-deficient mtDNA polymerase, *POLγ* ^exo−^ (equivalent to *PolgA* ‘mutator’ mice, above), revealed that excessive rather than insufficient autophagy contributes significantly to phenotypes caused by accumulating mtDNA mutations [121]. Curiously, reduction in *parkin* did not alleviate *POLγ* ^exo−^ phenotypes (homozygote larval lethality), indicating that the suppression did not involve Pink1/parkin mitophagy. The precise reasons why chronic activation of autophagy seems to become detrimental in this context are still unclear, but understanding when autophagy/mitophagy is beneficial versus detrimental for mitochondrial genome stability will be crucial for developing therapeutic strategies targeting mitochondrial diseases and age-related neurodegeneration.

## The role of PINK1/Parkin in immunity and inflammation

Neuroinflammation has long been recognised as a key component of PD pathophysiology, with microgliosis and proinflammatory cytokines often reported in patient samples [122,123]. Mechanistically, both innate and adaptive immunity have been implicated, with GWAS studies linking the human leukocyte antigen (HLA) region to idiopathic PD, and epidemiological studies indicating a protective effect of anti-inflammatory drugs [124,125]. The nature and origin of the initial inflammatory trigger(s) that may initiate or exacerbate neurodegeneration in idiopathic PD remain unclear, but *Drosophila* models hold much potential for helping elucidate this. Despite lacking a classical adaptive immune system, *Drosophila* have contributed fundamental insights into the mechanisms of innate immune signalling, including defining the NF-κB/Toll, NF-kB/IMD, JAK-STAT and JNK pathways [126]. Moreover, early transcriptomic studies in *Drosophila parkin* mutants revealed up-regulated innate immune pathways [127], which was subsequently also observed in *Pink1* mutants [85].

Given their proto-bacterial origin, mitochondria contain numerous immune-stimulatory components collectively referred to as mitochondrial damage-associated molecular patterns (mtDAMPs) [128]. Normally sequestered by the dual membrane structure of the mitochondria, disrupted MQC mechanisms can compromise mitochondrial integrity leading to the release of mtDAMPs into the cytosol, where they engage a range of pattern recognition receptors (PRRs) to induce aberrant immune activation. As the most extensively studied mtDAMP, mtDNA is known to activate several innate immune pathways when exposed in the cytosol, including the cGAS-STING, NLRP3 inflammasome and TLR pathways [129].

In recent years, a number of exciting *in vivo* studies have implicated mammalian PINK1/Parkin in both innate and adaptive immune pathways. Investigating mechanisms of mitochondrial antigen presentation (MitAP), PINK1 and Parkin were found to actively inhibit MitAP by suppressing the formation of MDVs [130]. Oral infection of *Pink1* KO mice with gram-negative bacteria led to an up-regulation of MitAP and the formation of brain-infiltrating, mitochondria-reactive CD8+ T cells. This caused pathological locomotor defects and DA neuron abnormalities [131]. These results suggest a potential autoimmune component linked to PINK1/Parkin dysfunction and also implicate the gut as an important site of inflammatory stimulation (see below). A parallel study, extending the previously described synergism between *Pink1/Prkn* KO and *PolgA* mtDNA ‘mutator’ mice that provoked locomotor deficits and DA neurodegeneration, found elevated proinflammatory cytokines and circulating mtDNA in these mice [132]. Similar effects were seen when *Prkn* KO mice were subjected to exhaustive exercise, to provoke an acute mitochondrial stress. Strikingly, genetic ablation of *STING* prevented the inflammatory response and completely abrogated locomotor defects and DA neurodegeneration, implicating the mtDNA-mediated cGAS-STING activation as the cause of neuroinflammation and neurodegeneration. However, the validity of these results has recently been cast into doubt with the full retraction of this study and independent replication yet to emerge [133]. Nevertheless, these and other studies suggest that PINK1 and Parkin likely influence immune regulation, prompting investigations using the *Drosophila* models.

Although the absence of a classical adaptive immune system limits its capacity to investigate mechanisms such as PINK1/Parkin-mediated suppression of MitAP, *Drosophila* have a homologous pathway to cGAS-STING (cGLR-Sting), which activates the NF-κB homologue, Relish, motivating analysis of their involvement in *Pink1/parkin* phenotypes [134,135]. An initial study found a lack of genetic interaction between *Pink1/parkin* and either *Sting* or *Relish*, with loss of *Sting* or *Relish* failing to suppress any *Pink1/parkin* phenotypes [136]. In fact, loss of *Relish* worsened *Pink1* mutant viability. This was surprising at the time given the earlier link between STING and PINK1/Parkin from the mouse study [132], but warrants re-evaluation. In contrast, a subsequent study in flies reported that *Sting* KO partially ameliorates neuromuscular phenotypes in *parkin* mutants with modest effects on *Pink1* mutants [137]. This discrepancy was attributed to differences in genetic background but requires clarification as other factors such as environment and microbiome may also be confounding. Nevertheless, the underlying mechanisms of suppression were suggested to involve oxidative stress and apoptosis, representing non-canonical roles of Sting. A further study proposed Relish as the putative regulator of the transcriptional signature of aberrant immune activation in *Pink1* mutants [138] and presented some evidence that partial loss of *Relish* suppressed sleep disruption and DA neurodegeneration [136]. While this study did not analyse Sting, they suggested that *eya*, a putative cytosolic DNA sensor, could be the upstream activator of Relish [139]; however, the evidence for eya as a cytosolic DNA sensor is currently rather limited. Clearly, the robustness of any potential interaction between Sting, NF-κB/Relish and Pink1/parkin is very uncertain and requires more stringent validation, especially in light of the retraction of the mammalian work.

Interestingly, the *Relish/eya* study also investigated the role of *Pink1*-related immune activation in the fly gut – a crucial organ for immune activation in flies [138]. The GI tract has emerged as a compelling site of origin for the initial inflammatory events contributing to PD [140]. Early studies led Braak and colleagues to propose the ‘ascending hypothesis’ by which misfolded α-Synuclein originates in the enteric nervous system and propagates to the central nervous system (CNS) via the vagus nerve [141]. Since then, accumulating clinical and experimental evidence has increasingly supported a contributory role of the gut-brain axis in PD pathogenesis [142,143]. Moreover, the immune system has emerged as a critical mediator of this bidirectional communication. Evidence has demonstrated that inflammatory events originating in the gut can profoundly influence CNS function via cytokine signalling or microbial metabolites, and increased PD risks have been found in individuals with chronic GI inflammation. Given the conserved physiological and anatomical features of the *Drosophila* GI tract [144], the underlying mechanistic links between both inflammation and the axis and PD could be effectively addressed using *Drosophila.*

Fedele et al. implicated the gut as a critical site of Relish-mediated inflammation driving neurodegeneration, as knockdown of *Relish* in the midgut appeared to ameliorate both intestinal and neurodegenerative phenotypes in *Pink1* mutants [138]. However, interpreting such a gut-to-brain inter-organ communication relies on absolute selectivity of the intended ‘tissue-specific’ tools used. This study used the NP3084-GAL4 ‘midgut’ driver to induce ‘gut-specific’ knockdown of *Relish*. However, while NP3084-GAL4 does express in the midgut, it also expresses in several neurons in the larval and adult brain [145]. While this is a useful tool for addressing tissue-autonomous effects in the gut, the neural expression undermines the interpretation of any gut-brain axis effects, leaving the precise contribution of gut-derived immune activation in *Pink1* mutants unresolved. Nevertheless, Fedele and colleagues did present data indicating GI tract abnormalities in the *Pink1* mutants, which is ripe for further investigation. Although currently limited, emerging evidence of intestinal dysregulation in *Pink1/parkin* mutants underscores the potential involvement of the gut-brain axis in Pink1/parkin-related pathology which is highly tractable in these models [138,146,147]. Notably, both Pink1 and parkin have been implicated as key regulators of gut homeostasis in *Drosophila*, exerting roles in both developmental and adult stages via the clearance of mitochondria [45] or, curiously, endoplasmic reticulum (ER) [44], as well as the modulation of intestinal stem cell proliferation [148].

## The role of (mitochondrial) calcium in PINK1/Parkin pathology

A critical question in trying to understand a disease such as PD is what accounts for the relative cell-type selectivity to the pathogenic process. Several unusual anatomical and physiological features of SNpc DA neurons, such as extensive arborisation and autonomous pacemaking activity supported by Ca_V_1.3 channels and lack of calcium-buffering proteins, are thought to contribute to the selective vulnerability of these neurons. In this context, disrupted Ca^2+^ homeostasis has long been implicated, but links to PINK1/Parkin function and dysfunction have been rather underexplored [149,150].

Ca^2+^ serves as a universal second messenger regulating numerous cellular functions and is particularly important in neurons, where it controls signal transmission and synaptic activity; hence, effective buffering and homeostatic regulation are essential. Following large influxes during neuronal firing, cytosolic Ca^2+^ is typically returned to low levels by ATP-dependent mechanisms, either as efflux through plasma-membrane pumps or uptake into the ER [151]. To a lesser extent, mitochondria also buffer cytosolic Ca^2+^, which contributes to overall cytosolic Ca^2+^ dynamics [152]. In addition to helping buffer cytosolic Ca^2+^, the uptake of Ca^2+^ into mitochondria can boost ATP production via Ca^2+^-sensitive TCA cycle enzymes [153]. The coupling of enhanced ATP production to areas of high cytosolic Ca^2+^ is augmented by the positioning of mitochondria through transport mechanisms mediated by the Ca^2+^-sensitive adaptor protein Miro1 (*RHOT1*) [154]. These mechanisms co-ordinate to boost mitochondrial activity at locations where high ATP levels are needed to restore appropriate Ca^2+^ levels and avoid catastrophic consequences of dysregulated Ca^2+^ signalling.

Disrupted Ca^2+^ homeostasis can lead to elevated cytosolic Ca^2+^ levels causing mitochondrial hyperpolarisation and increased production of ROS [150,155], placing additional strain on cellular defence mechanisms, including MQC. Moreover, excessive levels of mitochondrial Ca^2+^ (mCa^2+)^ can trigger the opening of the mitochondrial permeability transition pore and cell death [156]. The main mechanism of mCa^2+^ uptake is via the Mitochondrial Calcium Uniporter (MCU) complex located in the IMM, components of which are highly conserved in *Drosophila* [157]*.* The MCU complex requires relatively high levels of Ca^2+^ to be activated. This is achieved by localised release of Ca^2+^ from the ER, via inositol trisphosphate receptor (IP_3_R) or ryanodine receptor (RyR) channels, at sites where the two organelles come into close contact, so-called mitochondria-ER contact sites (MERCS). MERCS are complex, dynamic structures that serve multiple cell-signalling and metabolic purposes [158], orchestrated by a number of multi-protein complexes including the IP_3_R-GRP75-VDAC complex, that mediates ER-to-mitochondria Ca^2+^ transfer, as well as tethering by ER- and OMM-localised Mitofusins (an early-identified Parkin substrate)*.*

The intersection of Ca^2+^ flux and mitochondrial homeostasis presented a tantalising mechanistic link to PINK1/Parkin-related pathologies, and recent years have seen a growing connection between PINK1/Parkin function and (mitochondrial) Ca^2+^ homeostasis. Indeed, early studies analysing mechanisms of *PINK1*-related neuron vulnerability revealed that *PINK1* deficiency caused sensitisation to Ca^2+^-induced cell death via mCa^2+^ overload [159]. A parallel study extending the contemporary connections to mitochondrial fission/fusion dynamics showed that PINK1 affected mitochondrial trafficking via phosphorylation of Miro1 [46]. Subsequent studies linked the mCa^2+^ overload to disruption of the mCa^2+^ efflux channel, NCLX [160], which is modulated by PINK1-regulated protein kinase A (PKA) [161]. With the growing interest in the physiological importance of inter-organelle contact sites, coupled with the long-standing links between ER, mitochondria and Ca^2+^ dysfunction in PD, a slew of studies began to connect the alteration of MERCS structure and function to multiple PD-related proteins, including α-Synuclein, LRRK2 and DJ-1 (reviewed in [162]). Notably, the recognition of Mitofusins (Mfn1/2) as both a MERCS tether and a conserved Parkin substrate led to several *in vitro* studies reporting effects of Parkin on mitochondrial-ER tethering and Ca^2+^ transfer, but results have been rather inconsistent. Some *in vitro* studies reported loss of PINK1/Parkin activity increases MERCS [163,164] while others suggest it reduces them [165,166]. These seemingly discrepant results may be explained by cellular context or technical differences or even the methodologies used to analyse MERCS [167]. But ultimately, they do not address the impact on DA neurodegeneration or other physiological outcomes. Consequently, genetic and functional studies in *Drosophila* have helped to illuminate the impact of Pink1/parkin on mCa^2+^ homeostasis, and *vice versa*, *in vivo*.

Several studies have shown increased MERCS in *Drosophila Pink1/parkin* mutant neurons [168,169] and elevated basal mCa^2+^ levels [169,170]. Importantly, genetic reduction of MERCS proteins, whether tethering proteins such as Marf, ER proteins including the IP_3_R orthologue Itpr, or mitochondrial proteins such as VDAC/Porin or MCU, all reduce elevated mCa^2+^ levels and suppress *Pink1/parkin* mutant phenotypes including DA neurodegeneration, muscle degeneration, locomotion and lifespan [28,166,168–172]. Similarly, loss of Miro also suppressed *Pink1* mutant mCa^2+^ overload and DA neuron loss [170]. While the lack of robust phenotypes in murine models has precluded equivalent analysis to date, the inhibition of *MCU* was shown to suppress DA neurodegeneration in *pink1* ^−/−^ zebrafish [173], supporting a conserved effect and potential therapeutic benefit.

An exciting recent development has indicated a more direct link between PINK1/Parkin and Ca^2+^ signalling by impinging on ER Ca^2+^ release. Ham and colleagues found that the loss of *PINK1/Parkin* caused increased ER Ca^2+^ release in both mammalian cells and flies. This could be prevented by reducing the levels of the Parkin substrate CISD1 (also known as mitoNEET) and its fly counterpart Cisd which accumulates in flies upon loss of *Pink1/parkin* [172]. CISD1/Cisd is a small OMM-resident, 2Fe-2S cluster-containing protein with multiple roles in iron and ROS homeostasis [174], which has been known as a Parkin substrate for some years [51,175,176]. Mechanistically, CISD1/Cisd physically interacted with ER-localised IP_3_R, presumably at MERCS, increasing its activity and causing excess ER Ca^2+^ release. Genetic and pharmacological inhibition of CISD1/Cisd suppressed not only the aberrant ER Ca^2+^ release in cells and flies, but also suppressed *Pink1/parkin* phenotypes, including locomotor activity, DA neurodegeneration and mitochondrial integrity *in vivo* [172]. Despite the implications of ER Ca^2+^ release on mitochondrial Ca^2+^ uptake, the impact of CISD1/Cisd dysregulation on mCa^2+^ handling is currently unknown. Nevertheless, this discovery offers a novel insight into potential pathogenic mechanisms.

In a fascinating development, two additional studies, also combining fly and cell experiments, similarly reported CISD1/Cisd as a major contributor to PINK1/Parkin pathologies [47,177]. Both studies showed that genetic reduction of the accumulated parkin substrate Cisd substantially suppressed *Pink1* and *parkin* phenotypes *in vivo*, in agreement with Ham et al.; however, the mechanistic investigations highlighted quite different pathways. Martinez et al. showed that aberrant accumulation of Cisd, previously shown to accumulate with normal ageing and affect mitochondrial morphology in *Drosophila* [178]*,* blocked mitophagy by inhibiting autophagic flux [47]. Consequently, genetic reduction of CISD1/Cisd was sufficient to up-regulate mitophagy *in vitro* and *in vivo*. While the mitophagy induction *in vivo* was Pink1/parkin-independent, it remains to be determined if this is the case *in vitro* or, indeed, which pathway(s) may mediate this. In contrast, Bitar and colleagues focused on the Fe-S cluster-binding capacity of CISD1/Cisd and showed that in flies or patient-derived DA neurons lacking *PINK1,* CISD1/Cisd proteins showed increased homodimerisation and reduced Fe-S cluster binding, leading to dysregulated iron homeostasis and oxidative stress [177]. Although these studies present disparate mechanistic consequences of CISD1 dysregulation following loss of PINK1/Parkin, together they significantly underscore CISD1/Cisd as a potentially important target for correcting PINK1/Parkin pathologies which warrants further investigation.

While it is early days for understanding the relationship between CISD1/Cisd and its effects on Ca^2+^ signalling at MERCS or the control of mitophagy, the regulation of autophagy by Ca^2+^ has been extensively studied. Several autophagy regulators, such as CaMKKβ, AMPK, mTORC1 and others, are Ca^2+^-sensitive and respond to cytosolic Ca^2+^ changes to promote autophagy [179]. Indeed, a recent study showed that CaMKKβ responds to ER Ca^2+^ transients to promote phagophore initiation via FIP200 [180]. There are limited data assessing this phenomenon *in vivo*; however, a recent study of *Drosophila* intestinal stem cells has suggested that mCa^2+^ could also regulate IP_3_R activity through Ca^2+^ oscillations at MERCS, regulating autophagy through an AMPK-independent pathway [181]. Another interesting line of evidence linking Ca^2+^ regulation to PINK1/Parkin function showed that PINK1/Parkin-mediated mitophagy is affected by the Ca^2+^-sensitive phosphatase Calcineurin, and that expressing constitutively active Calcineurin suppresses locomotor deficits in *Pink1* mutant flies [182]. Calcineurin is a known regulator of the transcription factor TFEB, a key regulator of autophagy and lysosomal gene expression [183], which is itself regulated by nutrient, energy and Ca^2+^ signalling. Hence, there is much scope for autophagy and mitophagy to be regulated by Ca^2+^-mediated mechanisms which warrants further investigation.

## Future perspectives

As discussed, early characterisation of *Drosophila Pink1* and *parkin* models was instrumental in defining their roles in maintaining mitochondrial homeostasis and identifying some key features of the underlying cell biology and signalling pathway. A wealth of subsequent *in vitro* molecular and cell-biological studies has enormously advanced our understanding of the mechanisms of PINK1/Parkin biology, but questions remain about how this pathway works *in vivo*. It is unclear what physiological stimuli activate the pathway (i.e. PINK1), whether it is sufficiently activated by modest or transient reduction in the mitochondrial membrane potential, or whether other pathological stimuli also activate PINK1, and if so, how? While many studies have used a variety of manipulations that depolarise mitochondria to trigger PINK1 activity, PINK1/Parkin signalling can also be stimulated by unfolded protein stress [55,184], apparently without membrane depolarisation, which may also be relevant in flies [185]. However, whether activation by unfolded protein stress is, in fact, also triggered by localised or transient membrane depolarisation is currently unresolved. Interestingly, a recent genome-wide CRISPR/Cas9 screen described functionally diverse activators of PINK1-Parkin mitophagy which appear to converge on disrupting mitochondrial membrane potential as an activating mechanism [186]. Thus, it seems likely that loss of mitochondrial membrane potential may be the critical mitochondrial feature that PINK1 senses to trigger mitophagy in a physiological context. Nevertheless, much remains to be determined about where and when this may occur in cells and tissues that are relevant to PD.

To date, much emphasis has been placed on the wholesale degradation of mitochondria but growing evidence supports a more piecemeal turnover, via PINK1/Parkin or other mechanisms. There is much still to learn about the how such processes are orchestrated and for what purpose. Is this to remove a particular kind of damage, perhaps following an oxidative burst? Does this require localised membrane depolarisation? Are the principal targets proteins, lipids or nucleic acids? It will be highly relevant to determine the extent to which mtDNA mutations play a role in triggering PINK1-Parkin mitophagy or, *vice versa*, whether PINK1/Parkin indeed mitigate mtDNA mutations *in vivo*. Clearly, the potential involvement of the cGAS-STING pathway and/or mtDNA release needs urgent clarification, particularly considering recent contradictory studies. Again, the genetic tractability of *Drosophila* and the growing number of fly models of mtDNA mutations [36,116,121,187,188] provide a powerful system to address these questions.

It will also be important from a therapeutic standpoint to gain a deeper understanding of how alternative mitophagy pathways may be able to compensate when PINK1/Parkin are inactivated, whether by mutation or by age-related disrupting modifications [189–191]. As discussed, the potential to induce mitophagy is gaining considerable attention as a viable therapeutic intervention as it has potential application to a wide range of medical conditions [192]. The enthusiasm for developing mitophagy-promoting therapies in the context of NDs should be matched with equal drive to understand the complex integration of mitophagy with other homeostatic processes such as mitochondrial biogenesis. After all, excessive mitophagy could cause catastrophic loss of mitochondria; consequently, mitophagy is also being evaluated in the context of killing cancer cells [193]. While the intense interest in PINK1/Parkin-mediated mitophagy has opened a window into mitochondrial degradation as a MQC process, it has also revealed that mitophagy forms part of normal animal physiology such as organelle clearance during erythrocyte maturation or the developmental remodelling of intestines. This is an important consideration when targeting mitophagy therapeutically but also opens additional experimental paradigms for revealing new mechanisms/genes/proteins that are involved.

An area that has had limited investigation to date but requires investigating *in vivo* is the extent to which defects in MQC, including but not limited to mitophagy, can confer non-cell-autonomous effects. Indeed, the growing connection between PINK1/Parkin dysfunction and aberrant immune signalling and inflammation implicates the involvement of non-cell-autonomous or systemic effects in the pathogenic cause. With evidence of aberrant innate immune signalling in the *Drosophila Pink1/parkin* mutants, these models are well-placed to thoroughly analyse which of the several deeply conserved signalling pathways are involved. Moreover, the wide range of genetic tools available will be invaluable in dissecting tissue-specific involvement, identifying the critical tissues where defective mitophagy may stimulate immune signalling, and which tissues may be most sensitive to the downstream effects. An exciting focal point here is the potential involvement of the gut as a site of immune activation and implications for the gut-brain axis, where *Drosophila* represents an outstanding model system [194]. Owing to its relatively simple immune system and microbiota, *Drosophila* offers a powerful tool to dissect the fundamental mechanisms underpinning gut-brain communication and its relevance to PD. Early theories of a gut-brain axis in PD postulated the ‘ascending propagation hypothesis’ of α-Synuclein pathology [195]; however, recent studies have begun to investigate alternative mechanisms of gut-brain signalling, including those mediated by immune pathways and microbial metabolites [140,142]. Transgenic models of *Drosophila* expressing human α-Synuclein have yielded important insights into potential pathogenic mechanisms [196], but have not yet been systematically investigated in the gut-brain context, presenting a fruitful opportunity. Likewise, much is yet to be gleaned from the *Drosophila* models on the role of PINK1/Parkin in the gut-brain axis.

The emerging importance of Ca^2+^ dysregulation in *PINK1/Parkin* pathologies presents another important area for therapeutic investigation. *Drosophila* models have been influential in establishing pathogenic links between Pink1/parkin and Ca^2+^ dysregulation contributing to DA neurodegeneration. In the next phase, the genetic tractability of *Drosophila* could allow the dissection of the precise mechanisms with current evidence highlighting excess MERCS contributing to aberrant ER and mCa^2+^ flux. *Drosophila* studies identified a particularly intriguing molecular player in CISD1/Cisd, which likely disrupts cellular homeostasis at multiple levels including ER Ca^2+^ release, mitophagic flux, iron handling and Fe-S cluster metabolism. Given the mechanistic link between ER Ca^2+^ release and mitochondrial Ca^2+^ uptake, and the building significance of mCa^2+^ to PINK1/Parkin pathologies, it will be crucial to understand the impact of CISD1/Cisd dysregulation on mCa^2+^ handling.

Moreover, since CISD1/Cisd dysregulation was also shown to impinge on PINK1/Parkin-independent mitophagy, this raises the question of whether the suppressing effects seen by inhibiting CISD1/Cisd are more related to Ca^2+^ handling or mitophagy, or a combination of both. Indeed, whether mitophagy pathways are regulated by Ca^2+^ remains an area of active discussion [197]. Multiple Ca^2+^-responsive mechanisms are known to regulate both autophagy and mitochondrial dynamics. Not only do the Ca^2+^-responsive Miro proteins dictate mitochondrial transport dynamics and facilitate Parkin-mediated mitophagy [198], but the Ca^2+^-regulated kinase, CaMKIα, and phosphatase, Calcineurin, antagonistically modulate the activity of Drp1 to affect mitochondrial dynamics [199,200], while Calcineurin also regulates TFEB [201], a transcription factor and master regulator of autophagy and lysosomal biogenesis [183]. Other factors acting at the intersection of metabolic sensing and autophagy regulation, such as AMPK, are also Ca^2+^-regulated [202]. Hence, much remains to be understood about the intricate regulation of mitophagy by normal and abnormal Ca^2+^ signalling.

In summary, over the past couple of decades, *Drosophila* models of *Pink1/parkin* mutations have delivered fundamental insights into their conserved cellular functions. Studies in recent years have continued this trajectory, uncovering exciting new pathogenic mechanisms that may be critical to understanding *PINK1/PRKN* pathologies (Figure 2). Although already well-established, the ease of use and genetic tractability of the *Drosophila* models still have much to offer the field in providing critical insights into PINK1/Parkin function and the pathogenic consequences of their dysfunction *in vivo*.

## Open Access

This article has been published open access under our Subscribe to Open programme, made possible through the support of our subscribing institutions, learn more here: https://portlandpress.com/pages/open_access_options_and_prices#conditional

## Abbreviations

GWAS: genome-wide association studies
MQC: mitochondrial quality control
PD: Parkinson’s disease
SNpc: substantia nigra pars compacta
